# Diagnostic Dilemma: An Atypical Case of Astrocytoma in a Patient with Relapsing–Remitting Multiple Sclerosis

**DOI:** 10.3390/neurolint13020025

**Published:** 2021-06-03

**Authors:** Chantal Kahovec, Aman Saini, Michael C. Levin

**Affiliations:** 1 Saskatoon Multiple Sclerosis Clinic, Saskatchewan Health Authority, Saskatoon, SK S7K 0M7, Canada; Chantal.Kahovec@saskhealthauthority.ca; 2 Office of the Saskatchewan Multiple Sclerosis Clinical Research Chair, College of Medicine, University of Saskatchewan, Saskatoon, SK S7K 0M7, Canada; aman.saini@usask.ca; 3 Department of Medicine, Neurology Division, College of Medicine, University of Saskatchewan, Saskatoon, SK S7N 0X8, Canada; 4 Department of Anatomy, Physiology and Pharmacology, College of Medicine, University of Saskatchewan, Saskatoon, SK S7N 5E5, Canada

**Keywords:** astrocytoma, tumefactive, demyelination, multiple sclerosis

## Abstract

Distinguishing between tumefactive demyelinating lesions (TDLs) and brain tumors in multiple sclerosis (MS) can be challenging. A progressive course is highly common with brain tumors in MS and no single neuroimaging technique is foolproof when distinguishing between the two. We report a case of a 41-year-old female with relapsing–remitting multiple sclerosis, who had a suspicious lesion within the left frontal hemisphere, without a progressive course. The patient experienced paresthesias primarily to her right hand but remained stable without any functional decline and new neurological symptoms over the four years she was followed. The lesion was followed with brain magnetic resonance imaging (MRI) scans, positron emission tomography–computed tomography scans, and magnetic resonance spectroscopy. Together, these scans favored the diagnosis of a TDL, but a low-grade tumor was difficult to rule out. Examination of serial brain MRI scans showed an enlarging lesion in the left middle frontal gyrus involving the deep white matter. Neurosurgery was consulted and an elective left frontal awake craniotomy was performed. Histopathology revealed a grade II astrocytoma. This case emphasizes the importance of thorough and continuous evaluation of atypical MRI lesions in MS and contributes important features to the literature for timely diagnosis and treatment of similar cases.

## 1. Introduction

The concurrence of glioma and relapsing–remitting multiple sclerosis (RRMS) is uncommon. Multiple sclerosis (MS) is diagnosed by clinical and radiological criteria meeting the 2017 McDonald’s Criteria [1]. In patients with tumefactive MS (an atypical variant of MS with large isolated demyelinated plaque), differentiating between tumefactive demyelinating lesions (TDLs) and glioma can be complicated [2]. For instance, some atypical TDLs may resemble gliomas and conversely, early-stage gliomas may mimic MS. This diagnostic dilemma may lead to a delay in diagnosis and could potentially affect the long-term clinical outcomes in such patients. TDLs are usually defined as large (>2 cm) demyelinating lesions with/without mass effect, perilesional edema, or gadolinium enhancement, which could mimic brain tumors radiologically and clinically [2]. Brain magnetic resonance imaging (MRI) is a sensitive technique for depicting demyelinating lesions in MS patients, but when used alone it fails to provide an accurate diagnosis in many cases of atypical TDLs that mimic a tumor. The definitive diagnosis is often made only after a surgical biopsy or resection of the lesion. Clinical suspicion of a brain tumor in MS usually arises if a patient experiences a steady progression of symptoms or neurological deficits in the presence of a TDL. We report a patient without a progressive course, who met clinical and MRI criteria for the diagnosis of relapsing–remitting multiple sclerosis (RRMS), with one of the lesions initially described as tumefactive (a TDL was deemed more likely than a neoplasm based on brain MRI imaging), which was later found to be a primary diffuse astrocytoma (WHO grade II). The patient provided informed consent for the publication of this case report.

## 2. Case Presentation

**Figure 1:**
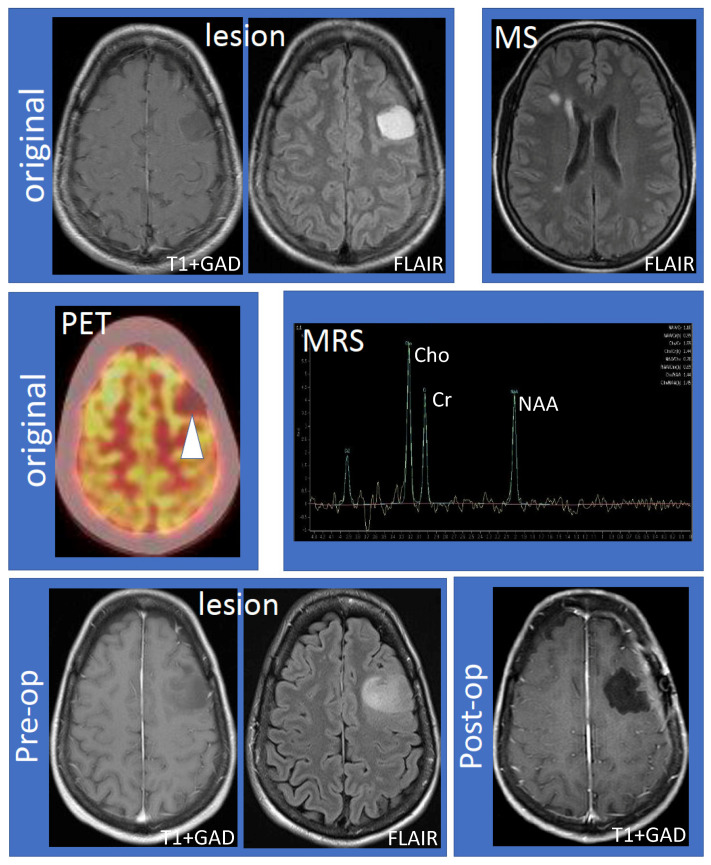
Work-up and progression of the left frontal lobe lesion. The top row shows the large, non-enhancing lesion distinct from MS lesions (left to right: T1 following gadolinium administration (T1 + GAD) and fluid attenuated inversion recovery (FLAIR) showing the lesion; FLAIR showing MS lesions). The middle row shows hypometabolism of the lesion using fluorodeoxyglucose positron emission tomography–computed tomography imaging and results of magnetic resonance spectroscopy (MRS). MRS values are as follows: N-acetylaspartate (NAA)/creatine (Cr) 1.10, NAA/Cr(h) 0.99, choline (Cho)/Cr 1.59, Cho/Cr(h) 1.44, NAA/Cho 0.70, NAA/Cho(h) 0.69, Cho/NAA 1.44, and Cho/NAA(h) 1.45. The bottom row shows that the lesion increased in size compared to the original MRI (“pre-op”) and successful resection of the lesion (“post-op”). Abbreviations: MS: multiple sclerosis; PET: positron emission tomography; MRS: magnetic resonance spectroscopy; Pre-op: pre-operative; Post-op: post-operative.

The 41-year-old female (non-smoker with a history of ulcerative colitis and celiac disease) had the onset of her MS 14 years ago, characterized by sensory symptoms of the lateral three fingers of the right hand, which resolved completely over several months. Seven years later, she experienced a second relapse characterized by paresthesias to her right hand, which spread to the medial aspect of her right arm, torso, leg, and toes. Following the second attack, she was diagnosed with RRMS based on MRI and clinical criteria. The initial MRI showed a large lesion (hypointense, non-enhancing on T1; hyperintense on fluid-attenuated inversion recovery (FLAIR) imaging) within the left frontal hemisphere involving the cortex and adjacent white matter (Figure 1, top row, “lesion”). Multiple white matter lesions were seen in both cerebral hemispheres compatible with MS (Figure 1, top row, “MS”). Focal hyperintensity was also observed in the right dorsal column of the cervical spinal cord at the C5–6 vertebral body level. Further evaluation by fluorodeoxyglucose positron emission tomography-computed tomography (FDG PET-CT) showed that the lesion was hypometabolic (Figure 1, middle row, “PET”). Magnetic resonance spectroscopy (MRS) revealed decreased N-acetylaspartate (NAA) with an NAA/creatine (Cr) ratio of 1.1 (Figure 1, middle row, “MRS”). The choline (Cho) was elevated, the Chol/Cr ratio was high at 1.59, and the Cho/NAA was 1.44. A significant lactate peak was not demonstrated. Neurosurgical and neuroradiological consultation suggested that the lesion in the left frontal hemisphere was consistent with a TDL in MS. Glatiramer acetate was initiated and the patient remained stable for the next four years without relapse or MRI activity. The patient had an expanded disability status scale (EDSS) of 1.0 since her diagnosis, without any evidence of confirmed disability progression or functional decline. The left frontal hemispheric lesion was followed over four years with serial MRI scans, which showed that the lesion increased in size with a mild mass effect involving the left middle frontal gyrus and underlying white matter (Figure 1, bottom row, “lesion”). There was interval stability of demyelinating plaques in the brain and cervical spinal cord. Neurosurgery was reconsulted and an elective left frontal awake craniotomy with the total resection of the lesion was carried out. Tissue diagnosis revealed primary left frontal diffuse astrocytoma (WHO grade II), 1p19q non-co-deleted, O6-methylguanine-DNA-methyltransferase (MGMT) methylated (46.5%), isocitrate dehydrogenase 1 (IDH1) R132H mutation, and loss of ATRX (alpha-thalassemia/mental retardation X-linked) expression. Post-operatively the patient experienced mild expressive dysphasia and was discharged on a tapering dose of dexamethasone. Following surgery, the patient received radiotherapy (54 Gray in 30 fractions) followed by temozolomide (6-cycle regime) for six months as an adjuvant treatment. She experienced a seizure, which was successfully treated with levetiracetam (750 mg) orally twice daily. She was on glatiramer acetate (20 mg) subcutaneously once daily throughout her course, and her MS remained stable by clinical and MRI criteria. Follow-up MRIs showed no recurrence of the glioma (Figure 1, bottom row, “post-op”) and stable white matter, ovoid, and periventricular lesions consistent with MS. 

## 3. Discussion

Differentiating between TDLs and brain tumors is challenging, particularly in the early stages of brain cancer. In this patient, diagnostic delay likely occurred because she was clinically stable, the lesion occurred in the setting of MS diagnosis (early in disease) and multiple imaging techniques could not adequately distinguish between TDL and early glioma. In addition to standard brain MRI imaging, the patient underwent FDG-PET-CT, which was hypometabolic (favoring TDL), and MRS, which was equivocal (Cho/NAA ratio = 1.44; >1.72 highly correlates with high-grade glioma [3]). The co-occurrence of glioma and MS in the same patient is uncommon, and most of the previously reported cases had high-grade astrocytic tumors that developed after MS diagnosis [4].

TDLs and brain tumors may share several characteristics, including size (>2 cm), gadolinium enhancement pattern, and a predilection for the frontal and parietal lobes. Importantly, several studies have attempted to distinguish between TDLs and brain tumors. For example, a study by Abdoli and Freedman [2] found that in contrast to TDLs, brain tumors in MS more commonly occurred in late or established MS, evolved over a few months, showed symptoms consistent with space-occupying lesions (cortical symptoms, raised intracranial pressure), had mostly a progressive course, and the lesion typically had more mass effect, necrosis, and perilesional edema and contained calcification or hemorrhage. Remarkably, the patient presented in this report had none of these findings.

Considering that there has been no recent review on this topic, we conducted an up-to-date topical review of the cases of gliomas in persons living with MS. A literature search was done on MEDLINE/PubMed, Scopus (Elsevier), and Google (internet search) using the following search terms: multiple sclerosis, glioma, tumefactive lesion, brain tumor, astrocytoma, ependymomas, and oligodendroglioma. Case reports or case series reporting gliomas in an individual with MS and published in the English language since 2012 were included. We found 14 articles published in the last eight years (2012–2020) (Table 1). There were 16 patients (seven females, nine males), of which 13 had RRMS and 3 had SPMS. Table 2 presents a brief summary and classification of the glioma cases identified in people living with MS. Fourteen tumors were located in the frontal and parietal lobes. The most commonly reported gliomas were glioblastomas/astrocytomas, followed by oligodendrogliomas. Importantly, in contrast to the patient presented in this report, the vast majority of the cases presented with a progressive course or new neurological symptoms. Unfavorable clinical outcomes were mostly observed in elderly patients with high-grade gliomas who were either in palliative care or had refused interventional procedures. Tumor recurrence was observed in two cases.

Low-grade glioma in an MS patient may pose a diagnostic dilemma as the MRI findings of low-grade gliomas may be similar to those of TDLs [11]. Table 3 highlights the characteristic features for the differential diagnosis of MS, TDLs, and brain tumors in MS. A high level of clinical suspicion in atypical TDLs is required to differentiate glioma from MS [12]. A stereotactic biopsy and histopathological examination of the lesion aids in making a definitive diagnosis in equivocal cases [19]. It is a reliable procedure and has a diagnostic accuracy of 82–99% [20]. Total resection of the tumor without inflicting additional neurological deficits typically offers better patient outcomes for long-term survival [21].

## 4. Conclusions

This case report highlights that slow-growing suspicious TDLs in MS patients with a non-progressive course should be carefully monitored by MRI over time to exclude low-grade gliomas. An elective craniotomy (with a total resection of the lesion) followed by radiation and chemotherapy may provide favorable outcomes in patients with concurrent RRMS and low-grade diffuse astrocytoma.

## Figures and Tables

**Table 1:** Topical review of recently reported cases of gliomas in MS patients.

Author, Year	Age/Sex	Age at MS Diagnosis	Age at Tumor Diagnosis	MS Phenotype	Tumor Location	Progressive Course or New Neurological Symptoms (Yes/No)	MRI Findings	Histopathology	Clinical Outcomes
Index Case (This Report)	41 y/F	34 y	41 y	RRMS	Left Frontal	No	Large, Non-Enhancing Lesion, T1 Hypointense, FLAIR Hyperintense	Primary Diffuse Astrocytoma (WHO grade II)	Post-Operative Mild Expressive Dysphasia and a Seizure. MS Remained Stable by Clinical and MRI Criteria
Turatti et al. [5], 2013	43 y/F	N.A.	43 y	RRMS	Splenium of corpus callosum and the optic radiations	Yes (progressive visual field restriction)	Large tumefactive lesion (>3 cm) that involved the spleniumof corpus callosum and the optic radiations, with moderate perilesional edema and without gadolinium enhancement	High-grade astrocytoma	Fully ambulatory, hyperreflexia with impaired vibration sensation and pain in the left leg, main disability: blindness caused by radiotherapy
Neil et al. [6], 2014	79 y/M	45 y	79 y	SPMS	Right parieto-occipital region	Yes (rapidly progressive cognitive decline)	Extensive, infiltrative mass which extended from the right parieto-occipital region across a significantly expanded corpus callosum	Anaplastic glioma	Family declined any further intervention and patient died in hospice
Carvalho et al. [7], 2014	43 y/M	23 y	37 y	RRMS	Left frontal lobe	Yes (progressive right hemiparesis)	Extensive subcortical and deep white matter lesion in the left frontal lobe (non-contrast-enhancing) on routine brain MRI	Grade II oligodendroglioma	At 3-year follow-up: stable MRI lesions with minor right hemiparesis and dysarthria
Mantero et al. [8], 2015	60 y/M	42 y	60 y	SPMS	Brainstem	Yes (rapidly progressing deterioration with tetraparesis, dysphagia, and dysarthria)	T2 hyperintense lesion in the brainstem, which increased in size on repeat MRI; thick enhancement surrounding a cyst-like cavity with involvement of left middle cerebellar peduncle	Glioblastoma	Patient died before the procedure (open brain biopsy); diagnosis was confirmed on autopsy
Morales et al. [9], 2017 Case 1	67 y/F	42 y	N.A	RRMS	Right frontoparietal lobe	Yes (had aggressive tumor and died 13 weeks after symptom onset)	Contrast-enhancing lesions in the right frontoparietal area which expanded in size and number over time	Glioblastoma multiforme with areas of necrosis and endothelial proliferation	Patient and family abstained from diagnostic work-up and patient died in hospice
Case 2	26 y/M	22 y	25 y	RRMS	Right superior frontal lobe	No (lesion was discovered in the routine surveillance MRI)	Right superior frontal lobe mass at the cortical surface	Diffuse astrocytoma,IDH-mutant, WHO grade II	Developed focal seizures after surgery (treated with levetiracetam); treated with natalizumab, stable for 5 years
	Same patient as above	22 y	26 y	RRMS	Right frontal lobe	Yes (developed new left-sided weakness)	Recurrence of the right frontal lobe lesion was observed on subsequent brain MRI	Glioblastoma multiforme with primary neuroectodermal tumor (PNET)-like component, IDH-mutant, WHO grade IV	Showed no evidence of tumor recurrence; continued monthly natalizumab and daily levetiracetam treatment for seizure prophylaxis
Kantorova et al. [10], 2017	27 y/F	19 y	28 y	RRMS	Right frontal lobe	Yes (progression of expanded disability status scale (EDSS) from 2.0 to 5.0 and development of new symptoms)	Atypical hyperintense tumefactive demyelinating lesion	Anaplastic astrocytoma	Remained unstable (frequent epileptic seizures) and died during status epilepticus before starting oncological treatment
Abrishamchi et al. [11], 2017	41 y/F	26 y	N.A.	RRMS	Left frontal lobe involving corpus callosum	Yes (progressive ataxia and dizziness)	High signal lesions in the left frontal lobe involving corpus callosum on FLAIR	Grade II astrocytoma	Impaired tandem gait, Romberg sign, bilateral Babinski signs, quadriparesis
Myserlis et al. [12], 2017	37 y/M	23 y	37 y	RRMS	Frontoparietal	Yes (gradual loss sensory and motor symptoms with cognitive decline)	A 5 cm circular, ring-enhancing lesion	Glioblastoma multiforme (grade IV)	Recurrence of the tumor with gradual loss of motor and sensory functions, ataxia, speech difficulties, cognitive decline and EDSS = 9
Preziosa et al. [13], 2017Case 1	59 y/M	43 y	59 y	SPMS	Right post-rolandic regions	Yes (gradual progression of spastic hypertonia, hyposthenia and EDSS)	T2-hyperintense and T1-hypointense pseudotumoral lesion in the right post-rolandic region with irregular and poorly defined margins	Glioblastoma with leptomeningeal infiltration (grade IV)	Deterioration of locomotor functions and inability to walk (possible consequences of radiotherapy). Patient refused diagnostic procedure
Case 2	55 y/M	38 y	53 y	RRMS	Left supero-anterior parietal regions	Yes (subtle difficulty in reading and fatigue)	Heterogeneous T2-hyperintense and T1-hypointense lesion in the left superior anterior parietal regions with internal cystic areas and irregular enhancement	Grade IV glioblastoma	Deterioration of cognitive function, fatigue, reading difficulty and acalculia (only radiotherapy and temozolomide were used; no surgical removal of lesion)
Shirani et al. [14], 2018	44 y/M	44 y	9 months after MS ds	RRMS	Right frontal lobe	Not evident (only the initial presentation of sudden onset right-sided optic neuritis was described)	Multiple FLAIR white matter lesions in periventricular, juxtacortical and subcortical areas	Grade II oligodendroglioma	An early postoperative brain MRI revealed a new demyelinating lesion in the right posterior periventricular white matter and the patient remained on glatiramer acetate at the time of the report.
Sinclair et al. [15], 2019	41 y/F	N.A.	30 y	RRMS	Left frontal lobe	Yes (gradually developed the clinical signs of mass-effect)	Suspected glioma in the left frontal lobe and multiple supratentorial MS-like lesions in both hemispheres	Oligodendroglioma; WHO grade 2–3	During the next 5 years after microsurgery, patient underwent systemic treatment due to a series of tumor- or MS-like relapses (signs of focal recurrence on postoperative MRI, scattered MS-suspected lesions, right-sided hemiparesis)
	Same patient as above	N.A.	39 y	RRMS	Left frontal lobe	Yes (right-sided hemiparesis)	Tumor regrowth was observed next to surgical site (deemed unsuitable for microsurgery)	Anaplastic astrocytoma	Patient suffered from right foot palsy from the second surgical resection and fatigue (due to chemotherapy)
Sirko et al. [16], 2020	30 y/M	31 y	32 y	RRMS	Right temporal lobe	Yes (a negative trend in the neurological examination)	T2 hyperintense lesion in the right temporal lobe and insula with irregular edges and blurred outlines involving both gray and white matter	Anaplastic oligoastrocytoma	Patient was waiting for surgery and was under the active supervision of a neurologist and a neurosurgeon
Algahtani et al. [17], 2020	23 y/F	23 y	23 y	RRMS	Left superior and part of the middle frontal gyri	Yes (gradually progressive walking difficulty and imbalance)	5.6 cm cortical-based tumor which originated from the left superior and part of the middle frontal gyri (surrounded by vasogenic edema)	Anaplastic oligodendroglioma (WHO grade III)	Six months post-surgery, new white matter demyelinating lesions consistent with MS were detected and diagnosis of RRMS was confirmed. Targeted sequencing revealed a mutation in the GBA2 gene consistent with the diagnosis of autosomal-recessive cerebellar ataxia with spasticity (positive family history)
London et al. [18], 2020	55 y/F	26 y	55 y	RRMS	Left frontal lobe	Yes (developed mild gait ataxia, right hypoesthesia, and nystagmus)	Closed-ring contrast-enhancing lesion in the left frontal lobe with surrounding edema	Glioblastoma (WHO grade IV)	Follow-up MRI 3 months post-operation showed tumor progression. Despite treatment, the patient worsened and she died 5 months after diagnosis

Abbreviations: M: male; F: female; N.A.: not available; y: year; MS: multiple sclerosis; RRMS: relapsing–remitting MS; SPMS: secondary progressive MS; IDH: isocitrate dehydrogenase; WHO: World Health Organization; MRI: magnetic resonance imaging; EDSS: expanded disability status scale; FLAIR: fluid attenuated inversion recovery.

**Table 2:** Summary and classification of the identified glioma cases in patients with MS.

Classification	Age (years)	Sex	MS Phenotype	Tumor Type [Case Reference]	Tumor Site
Glioblastoma/Astrocytoma (*n* = 10) Mean age = 47 years 5 M, 5 F Mostly in frontal or parietal lobes RRMS or SPMS	43	F	RRMS	Astrocytoma [5]	Splenium of corpus callosum and the optic radiations
	26	M	RRMS	Diffuse astrocytoma [9] Glioblastoma multiforme [9]	Right superior frontal Right frontal
	27	F	RRMS	Anaplastic astrocytoma [10]	Right frontal
	41	F	RRMS	Astrocytoma [11]	Left frontal lobe involving corpus callosum
	60	M	SPMS	Glioblastoma [8]	Brainstem
	67	F	RRMS	Glioblastoma multiforme [9]	Right frontoparietal
	37	M	RRMS	Glioblastoma multiforme [12]	Frontoparietal
	59	M	SPMS	Glioblastoma [13]	Right post-rolandic
	55	M	RMMS	Glioblastoma [13]	Left supero-anterior parietal
	55	F	RRMS	Glioblastoma [18]	Left frontal
Oligodendroglioma (*n* = 5) Mean age = 36.2 years 3 M, 2 F Frontal lobe RRMS	43	M	RRMS	Oligodendroglioma [7]	Left frontal
	44	M		Oligodendroglioma [14]	Right frontal
	41	F		Oligodendroglioma [15] Anaplastic astrocytoma [15]	Left frontal Left frontal
	30	M		Anaplastic oligodendroglioma [16]	Left superior and part of the middle frontal gyri
	23	F		Anaplastic oligodendroglioma [17]	Left superior and part of the middle frontal gyri
Undifferentiated (*n* = 1)	79	M	SPMS	Anaplastic glioma [6]	Right parieto-occipital

Abbreviations: M: male; F: female; MS: multiple sclerosis; RRMS: relapsing–remitting MS; SPMS: secondary progressive MS.

**Table 3:** Differential diagnosis of MS, TDLs, and brain tumors in MS.

	MS	TDLs	Brain Tumors in MS
Demographic features	Females > males; can occur at any age (usually between 20 and 40 years); relatively subacute onset [22]	Females > males; usually in young adults (middle age); relatively slow onset [23]	Brain tumors are more likely in males (this may vary with type of tumor); usually in older adults; gradual onset [24]
Clinical presentation	Variable, but typical syndromes include monocular loss of vision, double vision, ataxia, sensory loss, or limb weakness [25]	Polysymptomatic (usually sensory, motor, and cognitive symptoms), but may include focal neurological deficits, seizure, or aphasia [23]	Atypical manifestations may include (but are not limited to) headaches, tumor location-specific symptoms, or behavioral changes [26]
Clinical course	Relapsing–remitting (most common), secondary progressive, primary progressive	Could be monophasic or recurrent [23]	Usually progressive
Size and site of lesion(s)	3–5 mm or larger, typical white matter lesions in MS are periventricular, juxtacortical, and callososeptal, or at cerebellar peduncles in the infratentorial region [27]	>2 cm, often found in the supratentorial region (mostly in the frontal and parietal lobes) [28]	Large and variable size, usual distribution at frontal and temporal lobes [29]
MRI	Typical white matter lesions are round to ovoid in shape, should be at least 3 mm in size (long axis), and appear hyperintense on T2 and FLAIR sequences [30]	Large lesion (>2 cm) but with relatively little mass effect or surrounding edema, incomplete (open-ring pattern) contrast enhancement [28]	Mass effect, perilesional edema, necrosis, and continued enlargement of lesion [10]
H-MRS	Acute MS lesions: increased Cho, reduced NAA, and presence of lipids in acute MS lesions Chronic MS lesions: reduced NAA levels [31]	Increased Cho and lactate are supportive, but non-specific Typically demonstrates increased Cho/NAA ratio, reduced NAA/Cr ratio, and increased Cho/Cr ratio [23,32,33]	Persistently elevated Cho is more suggestive of tumor, could exhibit decreased NAA/Cr ratio, increased Cho/Cr ratio, and variable lactate and lipid peaks [13]

Abbreviations: MS: multiple sclerosis; TDLs: tumefactive demyelinating lesions; H-MRS: proton-magnetic resonance spectroscopy; mm: millimeter; cm: centimeter; Cho: choline; NAA: N-acetylaspartate; Cr: creatine; FLAIR: fluid-attenuated inversion recovery.

## Data Availability

The data used to support the findings of this case report are available within the article.

## Patient’s Perspective on the Treatment

From her initial diagnosis, they had found a rather large “MS lesion” in her left fronto-temporal region. A local neurologist determined that it was a large tumefactive lesion and referred her to the MS clinic for a second opinion. It was on her initial visit to the MS clinic in September 2017 that she heard the news that it might not be an MS lesion, but a low-grade glioma. The team at the clinic activated a referral process that ended with her having a craniotomy to remove the tumor. She is profoundly grateful to the team at the Saskatchewan MS Clinic for how they expedited her care. This team, she can say with wholehearted confidence, saved her life. She is also indebted to her neurosurgeons. Today she is optimistic about her prognosis and the progression of her disease.
